# 9-Allyl-9*H*-carbazole-3,6-dicarbaldehyde

**DOI:** 10.1107/S160053681203190X

**Published:** 2012-07-21

**Authors:** Bin Bin Hu, Xiang Chao Zeng, Lei Bian, Ru He

**Affiliations:** a Department of Chemistry, Jinan University, Guangzhou, Guangdong 510632, People’s Republic of China

## Abstract

In the title mol­ecule, C_17_H_13_NO_2_, the allyl group is almost perpendicular to the carbazole mean plane, with a dihedral angle of 89.0 (2)°. In the crystal, nonclassical C—H⋯O hydrogen bonds link the mol­ecules into corrugated sheets parallel to the *bc* plane. Weak inter­molecular π–π inter­actions are observed between the benzene rings [centroid–centroid distance = 3.874 (4) Å] from neighbouring sheets.

## Related literature

For applications of carbazole derivatives, see: Hong *et al.* (2012 ▶); Samanta *et al.* (2001 ▶); Koyuncua *et al.* (2011 ▶); Zhang *et al.* (2010 ▶). For related structures, see: Wang *et al.* (2008 ▶); Zhao *et al.* (2012 ▶).
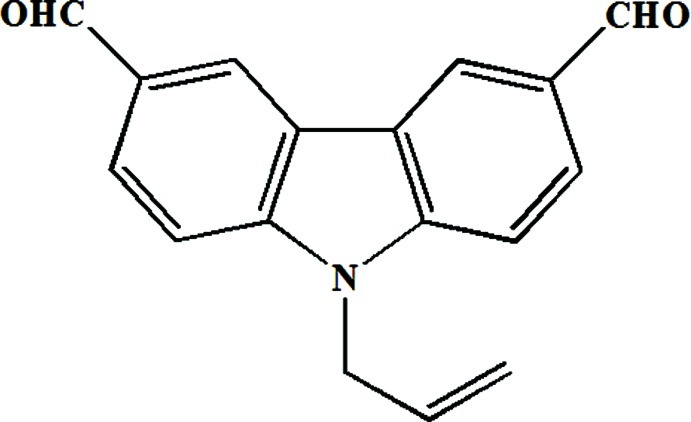


## Experimental

### 

#### Crystal data

C_17_H_13_NO_2_
*M* *_r_* = 263.28
Monoclinic, 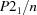

*a* = 8.4062 (8) Å
*b* = 10.3279 (10) Å
*c* = 15.2432 (19) Å
β = 94.958 (9)°
*V* = 1318.4 (2) Å^3^
*Z* = 4
Mo *K*α radiation
μ = 0.09 mm^−1^
*T* = 293 K
0.44 × 0.28 × 0.26 mm

#### Data collection

Oxford Gemini S Ultra area-detector diffractometer
Absorption correction: multi-scan (*CrysAlis PRO*; Oxford Diffraction, 2010 ▶) *T*_min_ = 0.963, *T*_max_ = 0.978
5464 measured reflections
2835 independent reflections
1909 reflections with *I* > 2σ(*I*)
*R*_int_ = 0.024

#### Refinement

*R*[*F* ^2^ > 2σ(*F* ^2^)] = 0.045
*wR*(*F* ^2^) = 0.118
*S* = 1.02
2835 reflections
182 parameters
H-atom parameters constrained
Δρ_max_ = 0.16 e Å^−3^
Δρ_min_ = −0.15 e Å^−3^

### 

Data collection: *CrysAlis PRO* (Oxford Diffraction, 2010 ▶); cell refinement: *CrysAlis PRO*; data reduction: *CrysAlis PRO*; program(s) used to solve structure: *SHELXS97* (Sheldrick, 2008 ▶); program(s) used to refine structure: *SHELXL97* (Sheldrick, 2008 ▶); molecular graphics: *SHELXTL* (Sheldrick, 2008 ▶); software used to prepare material for publication: *SHELXTL*.

## Supplementary Material

Crystal structure: contains datablock(s) I, global. DOI: 10.1107/S160053681203190X/cv5316sup1.cif

Structure factors: contains datablock(s) I. DOI: 10.1107/S160053681203190X/cv5316Isup2.hkl

Supplementary material file. DOI: 10.1107/S160053681203190X/cv5316Isup3.cml

Additional supplementary materials:  crystallographic information; 3D view; checkCIF report

## Figures and Tables

**Table 1:** Hydrogen-bond geometry (Å, °)

*D*—H⋯*A*	*D*—H	H⋯*A*	*D*⋯*A*	*D*—H⋯*A*
C2—H2⋯O1^i^	0.93	2.58	3.489 (3)	166
C5—H5⋯O2^ii^	0.93	2.54	3.332 (2)	143

Symmetry codes: (i) 
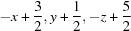
; (ii) 
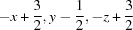
.

**Fig. 1.**
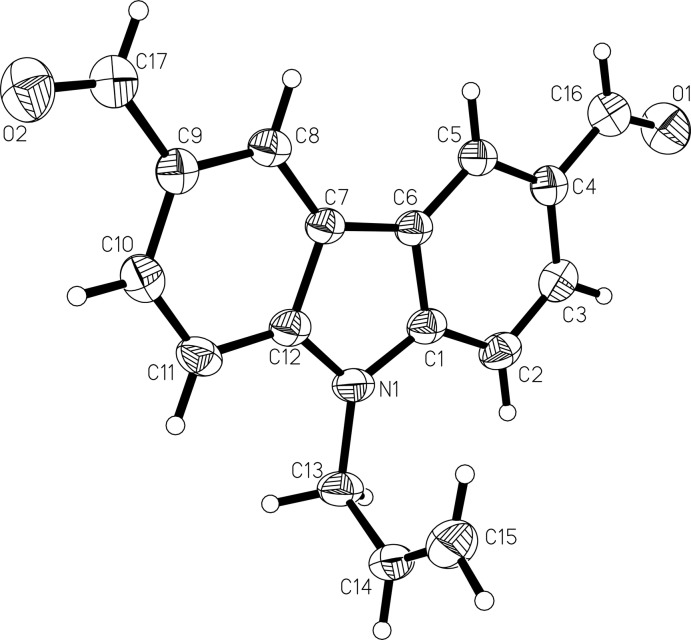
The molecular structure of the title compound, with the atom-numbering scheme. Displacement ellipsoids are drawn at the 30% probability level.

**Fig. 2.**
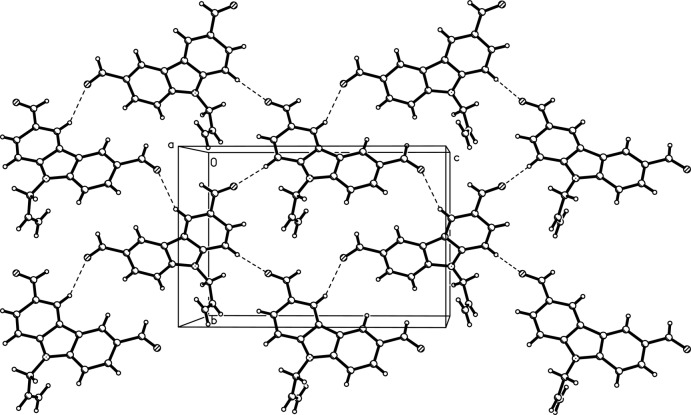
A portion of the crystal packing showing the sheet formed by the weak C—H···O hydrogen bonds (dashed lines).

## supplementary crystallographic information

### Comment

The carbazole ring has a highly conjugated π system with desirable optical and charge-transport properties, and these characteristics make carozole derivatives the excellent candidates to yield materials for applications in different areas of science, such as dye-sensitized solar cell (Hong *et al.*, 2012), electroluminescent (Samanta *et al.*, 2001), electrochromic displays (Koyuncua *et al.*, 2011) and antibacterial and antitumor agents (Zhang *et al.*, 2010). These are the reasons why they have attracted our interest. Here we report the crystal structure of the title compound which consists of a carbazole skeleton with a allyl group and two formacyls (Fig. 1).

In the title molecule, the bond lengths and angles are unexceptional, and generally agree with those observed in the related compounds (Wang *et al.*, 2008; Zhao *et al.*, 2012). The non-H atoms of the carbazole ring and the two formacyls are approximately coplanar with r.m.s. deviation from the best fit plane of 0.006 (3) °, the allyl group is almost perpendicular to the carbazole mean plane with a dihedral angle of 89.0 (2)°. In the crystal, C2—H···O1 and C5—H···O2 non-classical H-bonds (Table 1) link the molecules into corrugated sheets parallel to *bc* plane (Fig. 2). Weak intermolecular π–π interactions between the benzene rings [centroid-centroid distance = 3.874 (4) Å] from the neighbouring sheets stabilize further the crystal packing.

### Experimental

Phosphorus oxychloride (2.0 ml, 20 mmol) was added dropwise to the mixture of dry dimethylformamide (DMF, 3.0 ml, 40 mmol) and 9-allylcarbazole (2.07 g, 10 mmol) in chlorobenzene (20 ml) at 273 K under stirring. This solution was warmed up slowly to the room temperature in 0.5 h and stirred for another 0.5 h. After standing for 18 h at 343 K, more 3 ml DMF and 2 ml phosphorus oxychloride were added and stirred for 18 h continuously at the same temperature. After cooling, the resulting mixture was neutralized with saturated sodium bicarbonate solution until pH reached a value of 6 - 7, then the chlorobenzene was removed by water steam distillation, and the product was extracted with chloroform. After washing three times with water, the organic layer was dried over magnesium sulfate and evaporated *in vacuo*. The residue was separated by silica-gel column chromatography using petroleum ether-ethyl acetate (10:1) as eluting solvent and the title compound (I) was obtained (55.2% yield). Light brown crystals suitable for X-ray analysis (m.p. 429 K) grew over a period of one week when the ethyl acetate solution of I was exposed to the air at room temperature.

### Refinement

All H atoms were positioned geometrically [C—H = 0.97 Å for CH_2_, 0.93 Å for CH_2_(alkene), 0.93 Å for CH] and refined using a riding model, with *U*_iso_ = 1.2*U*_eq_ of the parent atom.

### Crystal data

**Table:**

C_17_H_13_NO_2_	*D*_x_ = 1.326 Mg m^−^^3^
*M**_r_* = 263.28	Melting point: 429 K
Monoclinic, *P*2_1_/*n*	Mo *K*α radiation, λ = 0.71073 Å
*a* = 8.4062 (8) Å	Cell parameters from 1300 reflections
*b* = 10.3279 (10) Å	θ = 3.3–29.4°
*c* = 15.2432 (19) Å	µ = 0.09 mm^−^^1^
β = 94.958 (9)°	*T* = 293 K
*V* = 1318.4 (2) Å^3^	Block, light brown
*Z* = 4	0.44 × 0.28 × 0.26 mm
*F*(000) = 552	

### Data collection

**Table:**

Oxford Gemini S Ultra area-detector diffractometer	2835 independent reflections
Radiation source: fine-focus sealed tube	1909 reflections with *I* > 2σ(*I*)
Graphite monochromator	*R*_int_ = 0.024
φ and ω scans	θ_max_ = 27.0°, θ_min_ = 3.3°
Absorption correction: multi-scan (*CrysAlis PRO*; Oxford Diffraction, 2010)	*h* = −6→10
*T*_min_ = 0.963, *T*_max_ = 0.978	*k* = −12→12
5464 measured reflections	*l* = −19→18

### Refinement

**Table:**

Refinement on *F*^2^	Secondary atom site location: difference Fourier map
Least-squares matrix: full	Hydrogen site location: inferred from neighbouring sites
*R*[*F*^2^ > 2σ(*F*^2^)] = 0.045	H-atom parameters constrained
*wR*(*F*^2^) = 0.118	*w* = 1/[σ^2^(*F*_o_^2^) + (0.0432*P*)^2^ + 0.2427*P*] where *P* = (*F*_o_^2^ + 2*F*_c_^2^)/3
*S* = 1.02	(Δ/σ)_max_ < 0.001
2835 reflections	Δρ_max_ = 0.16 e Å^−^^3^
182 parameters	Δρ_min_ = −0.15 e Å^−^^3^
0 restraints	Extinction correction: *SHELXL97* (Sheldrick, 2008), Fc^*^=kFc[1+0.001xFc^2^λ^3^/sin(2θ)]^-1/4^
Primary atom site location: structure-invariant direct methods	Extinction coefficient: 0.047 (4)

### Special details

**Table:**

Geometry. All e.s.d.'s (except the e.s.d. in the dihedral angle between two l.s. planes) are estimated using the full covariance matrix. The cell e.s.d.'s are taken into account individually in the estimation of e.s.d.'s in distances, angles and torsion angles; correlations between e.s.d.'s in cell parameters are only used when they are defined by crystal symmetry. An approximate (isotropic) treatment of cell e.s.d.'s is used for estimating e.s.d.'s involving l.s. planes.
Refinement. Refinement of *F*^2^ against ALL reflections. The weighted *R*-factor *wR* and goodness of fit *S* are based on *F*^2^, conventional *R*-factors *R* are based on *F*, with *F* set to zero for negative *F*^2^. The threshold expression of *F*^2^ > σ(*F*^2^) is used only for calculating *R*-factors(gt) *etc*. and is not relevant to the choice of reflections for refinement. *R*-factors based on *F*^2^ are statistically about twice as large as those based on *F*, and *R*- factors based on ALL data will be even larger.

### Fractional atomic coordinates and isotropic or equivalent isotropic displacement parameters (Å^2^)

**Table:**

	*x*	*y*	*z*	*U*_iso_*/*U*_eq_	
N1	0.59424 (16)	0.67631 (13)	1.01449 (10)	0.0497 (4)	
C7	0.69533 (17)	0.55686 (15)	0.90689 (11)	0.0421 (4)	
C5	0.84834 (18)	0.39928 (15)	1.01791 (11)	0.0451 (4)	
H5	0.8926	0.3478	0.9763	0.054*	
C1	0.68710 (19)	0.58033 (16)	1.05593 (12)	0.0461 (4)	
C12	0.59952 (19)	0.66450 (16)	0.92455 (12)	0.0461 (4)	
C9	0.64340 (19)	0.59883 (17)	0.75251 (12)	0.0483 (4)	
C6	0.75183 (18)	0.50343 (15)	0.99146 (11)	0.0422 (4)	
C4	0.87835 (19)	0.37238 (17)	1.10679 (12)	0.0483 (4)	
C8	0.71472 (19)	0.52431 (16)	0.82040 (11)	0.0455 (4)	
H8	0.7756	0.4525	0.8079	0.055*	
C2	0.7177 (2)	0.55521 (19)	1.14551 (12)	0.0552 (5)	
H2	0.6753	0.6072	1.1875	0.066*	
O1	1.00518 (18)	0.22217 (15)	1.20823 (10)	0.0826 (5)	
C11	0.5259 (2)	0.73978 (17)	0.85642 (13)	0.0537 (5)	
H11	0.4626	0.8105	0.8682	0.064*	
O2	0.62394 (17)	0.62669 (15)	0.59663 (10)	0.0806 (5)	
C17	0.6687 (2)	0.5651 (2)	0.66156 (13)	0.0587 (5)	
H17	0.7245	0.4891	0.6529	0.070*	
C3	0.8126 (2)	0.45114 (19)	1.16965 (12)	0.0555 (5)	
H3	0.8340	0.4321	1.2291	0.067*	
C14	0.5887 (2)	0.89280 (17)	1.08156 (13)	0.0592 (5)	
H14	0.5316	0.9572	1.1077	0.071*	
C10	0.5500 (2)	0.70602 (17)	0.77173 (13)	0.0546 (5)	
H10	0.5031	0.7556	0.7256	0.066*	
C13	0.5018 (2)	0.77164 (17)	1.05870 (13)	0.0573 (5)	
H13A	0.4682	0.7335	1.1122	0.069*	
H13B	0.4064	0.7923	1.0209	0.069*	
C16	0.9756 (2)	0.26040 (19)	1.13370 (14)	0.0601 (5)	
H16	1.0191	0.2138	1.0893	0.072*	
C15	0.7349 (3)	0.9192 (2)	1.06934 (15)	0.0763 (7)	
H15A	0.7975	0.8582	1.0435	0.092*	
H15B	0.7778	0.9992	1.0864	0.092*	

### Atomic displacement parameters (Å^2^)

**Table:**

	*U*^11^	*U*^22^	*U*^33^	*U*^12^	*U*^13^	*U*^23^
N1	0.0457 (8)	0.0427 (8)	0.0613 (10)	0.0001 (7)	0.0086 (7)	−0.0127 (7)
C7	0.0380 (8)	0.0360 (8)	0.0528 (11)	−0.0056 (7)	0.0068 (7)	−0.0053 (7)
C5	0.0409 (8)	0.0425 (9)	0.0527 (11)	−0.0054 (8)	0.0089 (7)	−0.0038 (8)
C1	0.0413 (8)	0.0424 (9)	0.0554 (11)	−0.0082 (8)	0.0087 (8)	−0.0086 (8)
C12	0.0397 (8)	0.0384 (9)	0.0605 (12)	−0.0079 (8)	0.0065 (7)	−0.0081 (8)
C9	0.0441 (9)	0.0470 (10)	0.0539 (11)	−0.0096 (9)	0.0047 (8)	0.0030 (8)
C6	0.0382 (8)	0.0398 (9)	0.0490 (10)	−0.0058 (8)	0.0069 (7)	−0.0058 (8)
C4	0.0445 (9)	0.0484 (9)	0.0524 (11)	−0.0071 (8)	0.0059 (8)	0.0036 (8)
C8	0.0432 (8)	0.0403 (9)	0.0541 (11)	−0.0025 (8)	0.0098 (7)	−0.0031 (8)
C2	0.0542 (10)	0.0593 (11)	0.0536 (12)	−0.0077 (10)	0.0141 (8)	−0.0134 (9)
O1	0.0964 (11)	0.0819 (10)	0.0701 (10)	0.0061 (9)	0.0108 (8)	0.0289 (9)
C11	0.0467 (9)	0.0379 (9)	0.0762 (13)	0.0018 (8)	0.0045 (9)	−0.0026 (9)
O2	0.0836 (10)	0.0966 (11)	0.0625 (10)	0.0006 (9)	0.0108 (8)	0.0236 (9)
C17	0.0551 (10)	0.0630 (12)	0.0588 (13)	−0.0077 (10)	0.0090 (9)	0.0089 (10)
C3	0.0556 (10)	0.0632 (12)	0.0481 (11)	−0.0115 (10)	0.0070 (8)	−0.0009 (9)
C14	0.0607 (11)	0.0458 (10)	0.0705 (14)	0.0025 (10)	0.0025 (10)	−0.0154 (9)
C10	0.0494 (10)	0.0454 (10)	0.0681 (13)	−0.0051 (9)	0.0000 (9)	0.0081 (9)
C13	0.0501 (10)	0.0510 (10)	0.0725 (13)	0.0022 (9)	0.0144 (9)	−0.0166 (10)
C16	0.0610 (11)	0.0576 (11)	0.0627 (13)	−0.0049 (10)	0.0105 (9)	0.0114 (10)
C15	0.0719 (14)	0.0600 (13)	0.0958 (18)	−0.0142 (12)	0.0002 (12)	−0.0126 (12)

### Geometric parameters (Å, º)

**Table:**

N1—C1	1.380 (2)	C2—C3	1.370 (3)
N1—C12	1.381 (2)	C2—H2	0.9300
N1—C13	1.455 (2)	O1—C16	1.208 (2)
C7—C8	1.384 (2)	C11—C10	1.369 (2)
C7—C12	1.412 (2)	C11—H11	0.9300
C7—C6	1.444 (2)	O2—C17	1.209 (2)
C5—C4	1.385 (2)	C17—H17	0.9300
C5—C6	1.386 (2)	C3—H3	0.9300
C5—H5	0.9300	C14—C15	1.288 (3)
C1—C2	1.392 (2)	C14—C13	1.475 (2)
C1—C6	1.409 (2)	C14—H14	0.9300
C12—C11	1.398 (2)	C10—H10	0.9300
C9—C8	1.384 (2)	C13—H13A	0.9700
C9—C10	1.403 (2)	C13—H13B	0.9700
C9—C17	1.463 (3)	C16—H16	0.9300
C4—C3	1.406 (3)	C15—H15A	0.9300
C4—C16	1.455 (3)	C15—H15B	0.9300
C8—H8	0.9300		
			
C1—N1—C12	108.99 (13)	C1—C2—H2	121.2
C1—N1—C13	125.26 (16)	C10—C11—C12	117.82 (16)
C12—N1—C13	125.72 (15)	C10—C11—H11	121.1
C8—C7—C12	119.27 (16)	C12—C11—H11	121.1
C8—C7—C6	134.49 (15)	O2—C17—C9	126.2 (2)
C12—C7—C6	106.23 (15)	O2—C17—H17	116.9
C4—C5—C6	119.54 (16)	C9—C17—H17	116.9
C4—C5—H5	120.2	C2—C3—C4	121.67 (17)
C6—C5—H5	120.2	C2—C3—H3	119.2
N1—C1—C2	129.20 (16)	C4—C3—H3	119.2
N1—C1—C6	108.83 (15)	C15—C14—C13	127.18 (19)
C2—C1—C6	121.96 (17)	C15—C14—H14	116.4
N1—C12—C11	129.66 (16)	C13—C14—H14	116.4
N1—C12—C7	109.06 (15)	C11—C10—C9	121.94 (17)
C11—C12—C7	121.27 (17)	C11—C10—H10	119.0
C8—C9—C10	119.79 (17)	C9—C10—H10	119.0
C8—C9—C17	119.17 (17)	N1—C13—C14	114.22 (14)
C10—C9—C17	121.04 (17)	N1—C13—H13A	108.7
C5—C6—C1	119.09 (16)	C14—C13—H13A	108.7
C5—C6—C7	134.05 (15)	N1—C13—H13B	108.7
C1—C6—C7	106.87 (14)	C14—C13—H13B	108.7
C5—C4—C3	120.10 (17)	H13A—C13—H13B	107.6
C5—C4—C16	119.06 (17)	O1—C16—C4	126.1 (2)
C3—C4—C16	120.82 (17)	O1—C16—H16	116.9
C7—C8—C9	119.89 (16)	C4—C16—H16	116.9
C7—C8—H8	120.1	C14—C15—H15A	120.0
C9—C8—H8	120.1	C14—C15—H15B	120.0
C3—C2—C1	117.63 (17)	H15A—C15—H15B	120.0
C3—C2—H2	121.2		
			
C12—N1—C1—C2	179.57 (16)	C6—C5—C4—C16	178.05 (14)
C13—N1—C1—C2	−2.3 (3)	C12—C7—C8—C9	1.4 (2)
C12—N1—C1—C6	−0.97 (17)	C6—C7—C8—C9	−179.34 (16)
C13—N1—C1—C6	177.21 (14)	C10—C9—C8—C7	−0.8 (2)
C1—N1—C12—C11	−179.46 (16)	C17—C9—C8—C7	178.34 (15)
C13—N1—C12—C11	2.4 (3)	N1—C1—C2—C3	178.68 (15)
C1—N1—C12—C7	1.20 (17)	C6—C1—C2—C3	−0.7 (2)
C13—N1—C12—C7	−176.96 (14)	N1—C12—C11—C10	−179.46 (16)
C8—C7—C12—N1	178.47 (13)	C7—C12—C11—C10	−0.2 (2)
C6—C7—C12—N1	−0.95 (17)	C8—C9—C17—O2	−174.17 (17)
C8—C7—C12—C11	−0.9 (2)	C10—C9—C17—O2	5.0 (3)
C6—C7—C12—C11	179.64 (14)	C1—C2—C3—C4	0.5 (3)
C4—C5—C6—C1	0.4 (2)	C5—C4—C3—C2	0.2 (3)
C4—C5—C6—C7	−179.03 (16)	C16—C4—C3—C2	−178.48 (16)
N1—C1—C6—C5	−179.23 (13)	C12—C11—C10—C9	0.8 (2)
C2—C1—C6—C5	0.3 (2)	C8—C9—C10—C11	−0.3 (3)
N1—C1—C6—C7	0.36 (17)	C17—C9—C10—C11	−179.48 (16)
C2—C1—C6—C7	179.87 (14)	C1—N1—C13—C14	91.2 (2)
C8—C7—C6—C5	0.6 (3)	C12—N1—C13—C14	−90.9 (2)
C12—C7—C6—C5	179.86 (16)	C15—C14—C13—N1	−2.6 (3)
C8—C7—C6—C1	−178.93 (16)	C5—C4—C16—O1	−176.38 (18)
C12—C7—C6—C1	0.36 (17)	C3—C4—C16—O1	2.3 (3)
C6—C5—C4—C3	−0.7 (2)		

### Hydrogen-bond geometry (Å, º)

**Table:**

*D*—H···*A*	*D*—H	H···*A*	*D*···*A*	*D*—H···*A*
C2—H2···O1^i^	0.93	2.58	3.489 (3)	166
C5—H5···O2^ii^	0.93	2.54	3.332 (2)	143

Symmetry codes: (i) −*x*+3/2, *y*+1/2, −*z*+5/2; (ii) −*x*+3/2, *y*−1/2, −*z*+3/2.
